# Investigation of the effects of urea cycle amino acids on the expression of *ALB* and *CEBPB* in the human hepatocellular carcinoma cell line FLC-4

**DOI:** 10.1007/s13577-020-00383-1

**Published:** 2020-05-30

**Authors:** Takahiko J. Fujimi, Yoshihiro Mezaki, Takahiro Masaki, Ayasa Tajima, Mariko Nakamura, Akira Yoshikawa, Noriyuki Murai, Mamoru Aizawa, Soichi Kojima, Yoshihiro Matsumoto, Hideki Aizaki, Tomokazu Matsuura

**Affiliations:** 1 Department of Registered Dietitians, Faculty of Health and Nutrition, Bunkyo University, Namegaya, Chigasaki, Kanagawa Japan; 2 Department of Laboratory Medicine, The Jikei University School of Medicine, Nishi-shinbashi, Minato-ku, Tokyo, Japan; 3 Department of Molecular Biology, The Jikei University School of Medicine, Nishi-shinbashi, Minato-ku, Tokyo, Japan; 4 Department of Applied Chemistry, School of Science and Technology, Meiji University, Higashimita, Tama-ku, Kawasaki, Japan; 5 Liver Cancer Prevention Research Unit, RIKEN Center for Integrative Medical Sciences, Hirosawa, Wako, Saitama Japan; 6 Department of Internal Medicine, Division of Gastroenterology and Hepatology, The Jikei University School of Medicine, Nishi-shinbashi, Minato-ku, Tokyo, Japan; 7 Department of Virology II, National Institute of Infectious Diseases, Toyama, Shinjuku-ku, Tokyo, Japan

**Keywords:** Albumin, Amino acid metabolism, Arginine, C/EBPβ, Nutrition, Ornithine

## Abstract

Cell lines are powerful tools for research into liver function at the molecular level. However, they are generally unsuitable for rigorously assessing the effects of amino acid composition, because many lines require serum-containing medium for their maintenance. Here, we aimed to investigate the effects of ornithine and arginine, which are included in the characteristic metabolic process in hepatocyte, on a human hepatoma-derived cell line (FLC-4) that can be cultured in serum-free medium. FLC-4 cells were cultured under the following three conditions: + ornithine/ – arginine, – ornithine/ – arginine, and –ornithine/ + arginine. Albumin expression evaluated by quantitative polymerase chain reaction and enzyme-linked immunosorbent assay and showed no obvious differences based on the presence of ornithine or arginine. However, the mRNA levels of two liver-enriched transcription factors (*CEBPB* and *HNF1A*), which are involved in regulating albumin expression, were significantly higher in cells grown in medium-containing arginine than that in cells grown in ornithine-containing medium. Western blotting showed that the levels both activating and inhibitory C/EBPβ isoforms were significantly increased in cells grown in arginine medium. Furthermore, we have found that depletion of both ornithine and arginine, the polyamine sources, in the medium did not cause polyamine deficiency. When ornithine and arginine were depleted, albumin production was significantly reduced at the mRNA level, *CEBPB* mRNA levels were increased, and the level of activating form of C/EBPβ was increased. The results of this study suggest that in hepatocyte, these two amino acids might have different functions, and because of which they elicit disparate cellular responses.

**Electronic supplementary material:**

The online version of this article (10.1007/s13577-020-00383-1) contains supplementary material, which is available to authorized users.

## Introduction

We had previously studied the development of bioartificial livers composed of a radial flow bioreactor (RFB) and human hepatocellular carcinoma-derived cell lines, FLC-4, FLC-5, or FLC-7 [1–4]. One of the major features of these cell lines is that they can be cultured in serum-free medium, meaning that studies can be performed without considering the amino acids present in serum. Our previous study showed that selection of serum-free medium is an important factor for effective use of bioartificial livers containing these cell lines [4]. In the study that used RFB and FLC-7 cells, ASF104N medium was shown to be more suitable for cell growth than the IS-RPMI medium (based on RPMI-1640). However, ASF104N was less suitable for producing fibrinogen, which is specifically produced in hepatocytes, than IS-RPMI. When comparing both media types, we had focused on the effects of two amino acids, ornithine and arginine, using media that had complementary compositions of ornithine and arginine (ASF104N: + ornithine/ − arginine; RPMI-1640: − ornithine/ + arginine). Similar results were observed with respect to albumin production in another experiment. Albumin production by FLC-4 cells was improved by replacing the ASF104N (+ ornithine/ − arginine) medium in the RFB with E-RDF (− ornithine/ + arginine) (Online Resource). Many commercially available media used for hepatocellular carcinoma cell culture contain arginine but not ornithine. Ornithine is used when culturing human-induced pluripotent stem cells (iPS cells), and is a key component of the hepatocyte selection medium. One particular medium composition (+ ornithine/ − arginine) helped to eliminate unwanted iPS cells from a culture of primary hepatocytes [5]. These observations suggest that the two amino acids, which appear to complement each other in the urea cycle, have differing effects on hepatocyte function.

Understanding the effects of the components of the medium on protein production by cultured cells is important for evaluating of hepatocellular carcinoma cell function. Albumin, which is produced specifically and in large amounts by hepatocytes, is an attractive model for evaluating these relationships. It is the most abundant serum protein and its physiological and clinical significance is well established [6]. Serum albumin levels are used as diagnostic/prognostic markers for various diseases, and as a nutritional assessment index in clinical practice. A plethora of reports exist regarding the factors regulating albumin synthesis [7–11]. Serum albumin concentration is maintained within a normal range by a combination of these various regulatory factors [12]. However, there is a general theory that hepatocyte-specific genes, including *ALB*, are usually regulated at the mRNA level by transcription factors enriched in the liver, including members of the C/EBP and hepatocyte nuclear factor (HNF) families [13]. To all of the various factors, it is not completely understood how signals controlling albumin expression are transduced in cells.

Ornithine and arginine are involved in the urea cycle, which is characteristic feature of hepatocytes. Citrulline is converted into arginine in hepatocyte cytoplasm, and arginine is subsequently hydrolyzed into urea and ornithine. Ornithine is used for the next reaction step along with carbamoyl phosphate, in the mitochondria. The citrulline produced in that reaction (in the cytoplasm) is converted into arginine [14]. Ornithine is also involved in polyamine synthesis as a source of putrescine, which is converted into spermidine and spermine [15]. The positive effects of ornithine on albumin synthesis were first reported by two research groups almost 30 years ago. The authors used polyamine synthesis inhibitors [α-difluoromethylornithine (DFMO) or α-methylornithine (MO)] to demonstrate that promotion of albumin synthesis by ornithine can occur through polyamines [7, 16, 17]. However, it is unclear whether inhibition of polyamine synthesis recapitulates ornithine deficiency, and any direct link between ornithine depletion and albumin expression has not been established. Therefore, in this report, the effects of ornithine and arginine on *ALB* expression were evaluated using FLC-4 cells and three variants of serum-free medium [(+ ornithine/ − arginine), (− ornithine/ − arginine), and (− ornithine/ + arginine)].

## Materials and methods

### Cell line, media, and culture conditions

The human hepatocellular carcinoma-derived cell line, FLC-4 (RRID: CVCL_D204), which was established and have been maintained at The Jikei University School of Medicine, was used in this study, as previous reports [1, 4, 18–20]. Cells were maintained in ASF104N serum-free medium (Ajinomoto Co., Inc., Tokyo Japan) at 37 °C in an atmosphere containing 5% CO_2_. The custom-made medium ASOR (−), which differ from ASF104N only with respect to ornithine [absent in ASOR (−)], was purchased from KOHJIN BIO Co., Ltd. (Saitama, Japan). The ASF104N medium includes ornithine (100 mg/L), but not arginine, which means that the amino acid components of the urea cycle are not present in ASOR (−). A third medium supplemented with arginine (200 mg/L) was also used in this study. The concentration of arginine in the medium was the same as that in RPMI-1640. In this study, we denoted the three medium conditions as “Orn” (= ASF104N), “Arg” {= ASOR (−) + Arg}, and “Dep (meaning depletion of both amino acids)” {= ASOR (−)}. The cells were seeded into 6-well plates at a density of 1 × 10^6^ cells/well in ASF104N, and after 3 days (day 3), the cells were treated with one of the three media {Orn, Arg, or Dep}. The medium was changed every 3 days, and cell counting and sampling (cells and culture supernatants) were performed on days 3, 6, and 12. For cell counting, only the cells that were not stained with Gibco™ Trypan Blue Solution (0.4%) (Thermo Fisher Scientific Inc., MA, USA) were counted.

### Polyamine quantification

High-performance liquid chromatography (HPLC) analysis was performed to determine polyamine levels in cultured cells. Cultured cells were lysed with TDT [25 mM Tris-HCl (pH 7.2), 1 mM EDTA, 0.01% Tween80] and sonicated using a BRANSON 250D (Branson Ultrasonics, CT, USA) (output 60%, 5 s × 3 times). The protein concentration in the lysate was measured using a DC™ Protein Assay Kit (Bio-Rad Laboratories, Inc., CA, USA), and then, the lysate was treated with HClO_4_ (final 4%). After centrifugation (13,000 *g*, 4 °C, 10 min), the filtered supernatant (using 0.45 µm PVDF membrane) was subjected to HPLC. HPLC-based polyamine quantification was performed in accordance with a previously described method [21].

### Quantification of ammonia and urea concentration

Cells were seeded into 6-well plates (*n* = 3) at a density of 1 × 10^6^ cell/well in ASF104N (day 0). After 3 days (day 3), the cells treated with one of the three media {Orn, Arg and Dep}, and on day 6, ammonium chloride (NH_4_Cl) was added to all media at a final concentration of 3 mM. At that time, control wells without any cells were prepared for each of the three media. Then, the medium was replaced with fresh one containing NH_4_Cl on day 9. Aliquots of fresh media after exchange (day 9) and old media (without exchange; day 12) were subjected to ammonia and urea quantification. Ammonia quantification was performed using a WAKO NH3 test (WAKO Pure Chemical Industries, Ltd., Osaka, Japan) at a reduced (12.5%) scale from the manufacturer’s protocol. Urea quantification was performed with the QuantiChrom Urea Assay Kit (BioAssay Systems, CA, USA), according to the manufacturer’s protocol. Numerical differences in optical absorbance (520 nm) between days 12 and 9, after subtracting the control values (without cells), were used for calculation.

### Quantitative real-time PCR

Total RNA from cultured cells was isolated using the Qiagen RNeasy mini kit (Qiagen, Hilden, Germany), and further purified with DNase I (Takara Bio Inc., Shiga, Japan) and TRIZOL^®^ reagent (Thermo Fisher Scientific Inc., MA, USA). Complementary DNA was synthesized from purified total RNA using a High-Capacity cDNA Reverse Transcription kit (Applied Biosystems™, Thermo Fisher Scientific Inc.) in accordance with the manufacturer’s protocol. Real-time quantitative PCR (qPCR) was performed using a TaqMan^®^ probe system. The TaqMan^®^ probe and primer sets were purchased from Applied Biosystems™ (Thermo Fisher Scientific Inc.) in the form of the TaqMan^®^ Gene Expression Assay System [GAPDH; Hs99999905_m1, *ALB* (Albumin); Hs00910225_m1, *HNFA* (HNF1α); Hs00167041_m1, *HNFB* (HNF1β); Hs01001602_m1, *HNF4A* (HNF4α); Hs00230853_m1, *CEBPA* (C/EBPα); Hs00269972_s1, *CEBPB* (C/EBPβ); Hs00270923_s1]. Thermal cycling reactions and data analyses were performed using a StepOnePlus™ Real-Time PCR System and the StepOne™ software Ver. 2.2.2 (Applied Biosystems™, Thermo Fisher Scientific Inc.). Data were analyzed via the 2^−ΔΔ*C*(*t*)^ method [22].

### Enzyme-linked immunosorbent assay (ELISA)

For albumin quantification, sandwich ELISA was performed using two antibodies against human serum albumin [0855029 (MP BIOMEDICALS, CA, USA), capture antibody; AB19181 (Abcam, Cambridge, UK), detection antibody]. The assay plate was coated with the capture antibody (500×) at room temperature (24‒28 °C) for 2 h and then blocked with 1% BSA prepared in PBS(−). Culture media were diluted 100-fold with the blocking reagent and used as assay samples. Samples were probed with the HRP-conjugated secondary detection antibody (5000×) at room temperature for 2 h. For the detection of albumin-antibody specific reactions, 0.3 mM 4-methylumbelliferyl phosphate was used as a fluorescent substrate for HRP (excitation wavelength: 360 nm, emission: 460 nm).

### Western blotting

Cultured cells were lysed using an RIPA Lysis Buffer System (Santa Cruz Biotechnology, TX, USA) in the presence of protease and a phosphatase inhibitors, in accordance with the manufacturer’s protocol. After quantifying protein concentration, cell lysates containing equal amounts of protein were boiled with 2 × sample buffer {0.125 M Tris-HCl (pH 6.8), 10% (v/v) 2-mercaptoethanol, 4% (w/v) Sodium dodecyl sulfate, 10% (w/v) sucrose, 0.01% (w/v) Bromophenol Blue}. These samples were loaded onto a SuperSep™ Ace (10%) polyacrylamide gel (Wako Pure Chemical Industries, Ltd.) for electrophoretic separation, and transferred onto a polyvinylidene fluoride membrane via TransBlot SD (Bio-Rad Laboratories, Inc.). The membrane was probed with antibodies against C/EBPβ (sc-150X; Santa Cruz Biotechnology) (RRID: AAB_2260363). Detection and imaging of the specific bands were performed using the ECL™ Prime Western Blotting Detection System (GE Healthcare UK Ltd., Buckinghamshire, UK) and ChemiDoc™ Touch imaging system (Bio-Rad Laboratories, Inc.). Band intensities were calculated using ImageJ 1.52a (RRID: SCR_003070) (National Institutes of Health, MD, USA).

### Statistical analysis

Quantification of data is presented as the mean ± standard deviation (*n* = 3). To evaluate the statistical difference between the three groups (Orn, Dep, and Arg) from data collected on the same day, one-way analysis of variance (ANOVA) and Tukey’s honestly significant difference (HSD) test were performed using VassarStats (https://vassarstats.net/). The threshold for statistical significance was set to *P* < 0.05. Tukey’s HSD test results are shown as *: *P* < 0.05 and **: *P* < 0.01.

## Results

### Polyamine synthesis and ammonia metabolism under three medium conditions

**Fig. 1:**
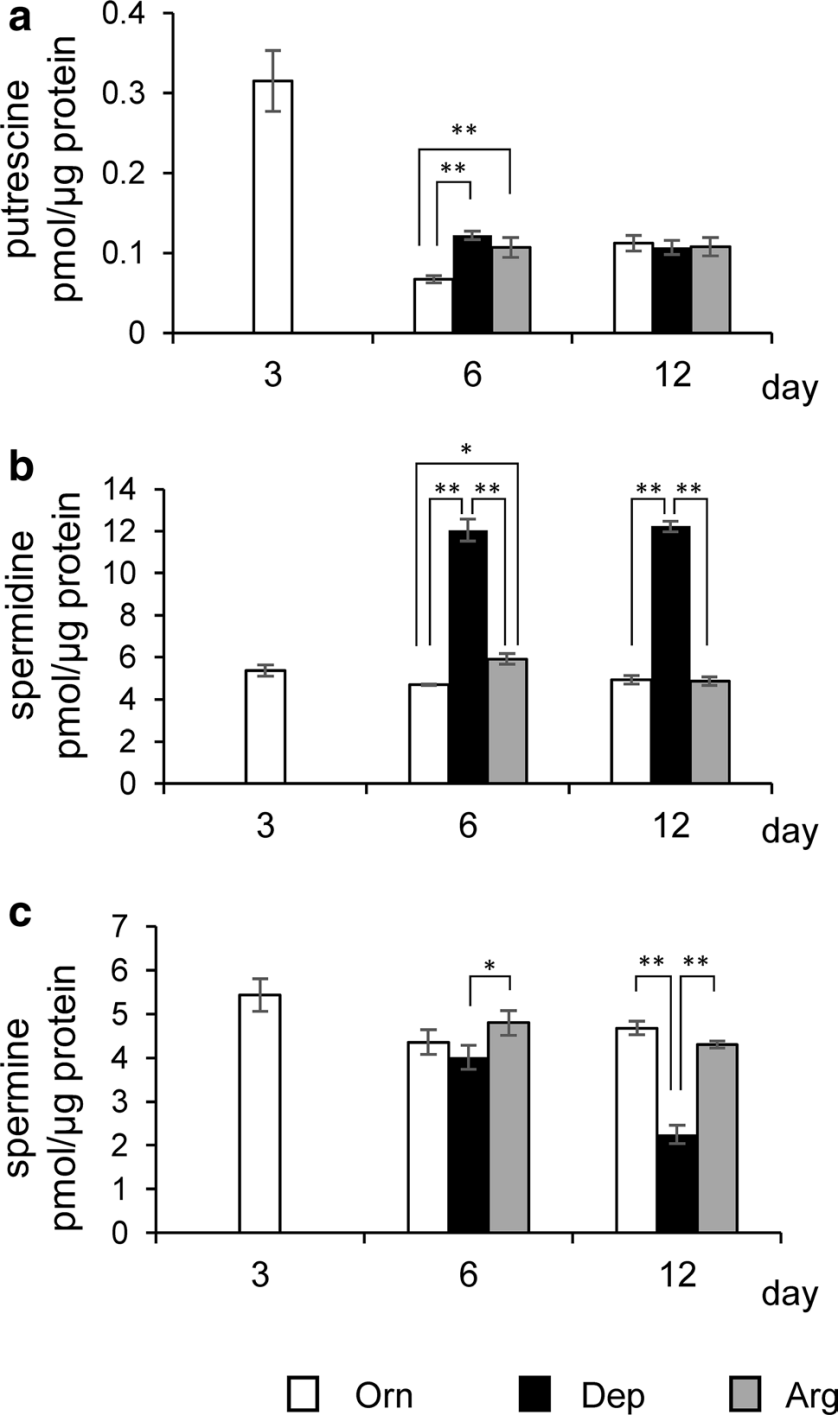
Quantitative analysis of polyamine levels in FLC-4 cells cultured under three different medium conditions. Three kinds of polyamine molecules metabolized from ornithine were quantified, i.e., putrescine (**a**), spermidine (**b**), and spermine (**c**). Polyamine molecules were extracted from FLC-4 cells cultured under each medium condition (white: Orn, black: Dep, gray: Arg) and quantified. All values in this figure are shown as the average of three samples (*n* = 3). A significant difference in putrescine level among the three groups was observed at day 6 (*F* = 34.39, *P* = 0.000517), but not at day 12 (*F* = 0.19, *P* = 0.831748). For spermidine and spermine, significant difference was observed on day 6 and day 12 {Spermidine: day 6; (*F* = 416.31, *P* < 0.0001), day 12; (*F* = 1403.25, *P* < 0.0001)} {Spermine: day 6; (*F* = 5.81, *P* = 0.039485), day 12; (*F* = 197.06, *P* < 0.0001)}. Asterisks indicate the results for comparisons between groups by Tukey’s HSD test (*P < 0.05, **P < 0.01)

As described above, there are several reports indicating a positive effect of ornithine on albumin production, as mediated by polyamines [7, 16, 17]. These have been shown in experiments with polyamine synthesis inhibitors, but it is unclear whether inhibition of polyamine synthesis mimics ornithine deficiency, and the direct link between ornithine deficiency and ALB expression remains unclarified. We first performed quantitative analyses of polyamine levels in FLC-4 cells cultured under three different medium conditions. These were performed to elucidate whether ornithine deficiency directly causes polyamine deficiency (Fig. 1). Interestingly, polyamine starvation was not observed in cells cultured in Dep medium that contained neither ornithine nor arginine. Conversely, only spermidine levels were increased, by approximately 2.5-fold, in Dep medium at both days 6 and 12, as compared to the other two medium conditions (Fig. 1b). When comparing Orn and Arg, the only significant differences found were for putrescine and spermidine levels at day 6 (Fig. 1a, b, respectively). From these results, we can reject the hypothesis that ornithine deficiency and inhibition of polyamine synthesis are the same condition.

**Fig. 2:**
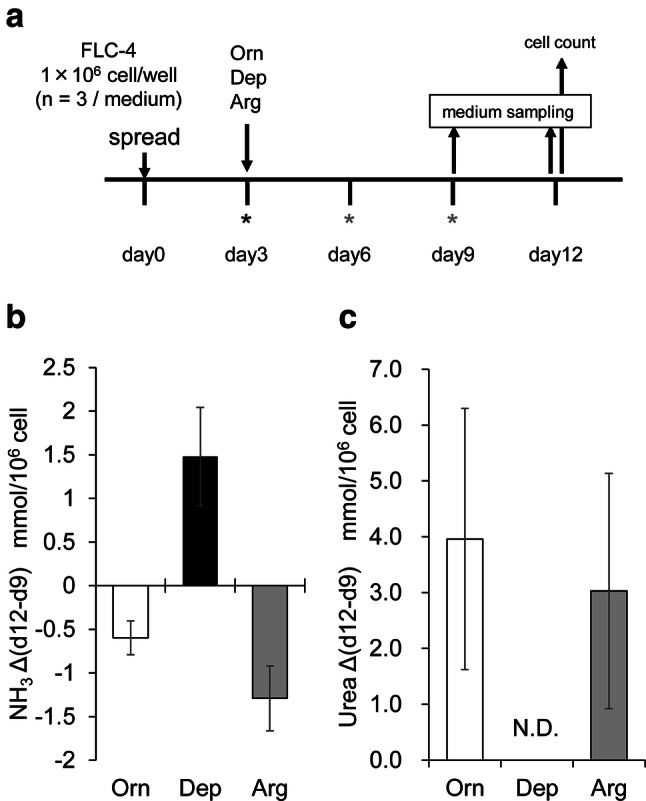
Quantitative analyses of ammonia and urea concentrations. **a** Schematic drawing of the experimental design for quantifying ammonia and urea concentrations. Asterisks indicate the points of medium change (black: media without ammonia chloride; gray: media containing ammonium chloride). **b** Quantification of differential amounts of ammonia between days 9 and 12 in three different medium conditions. Ammonia levels were normalized to cell number {NH_3_∆(d12–d9) μmol/10^6^ cells}. **c** Quantification of differential amounts of urea between days 9 and 12 in three different medium conditions. Urea levels were normalized to cell number {Urea∆(d12–d9)/10^6^ cells} N.D. indicates a non-detectable level

Our previous study using microarray analysis showed that the many ammonium metabolism genes were not expressed in FLC-4 cells cultured in RFB [19]. To determine whether the urea cycle is active under the culture conditions used in this study, urea and ammonia levels were evaluated (Fig. 2). Differences in ammonia concentration between days 9 and 12 showed that ammonia levels were decreased under Orn and Arg conditions, but not decreased under Dep condition (Fig. 2b). Urea production was also observed under Orn and Arg conditions, but could not be detected under the Dep condition (Fig. 2c). These results indicate that ammonia metabolism involving the urea cycle was active in FLC-4 cells when the medium contained either ornithine or arginine.

### Responses in FLC-4 cells with respect to growth and albumin production under three different culture conditions

**Fig. 3:**
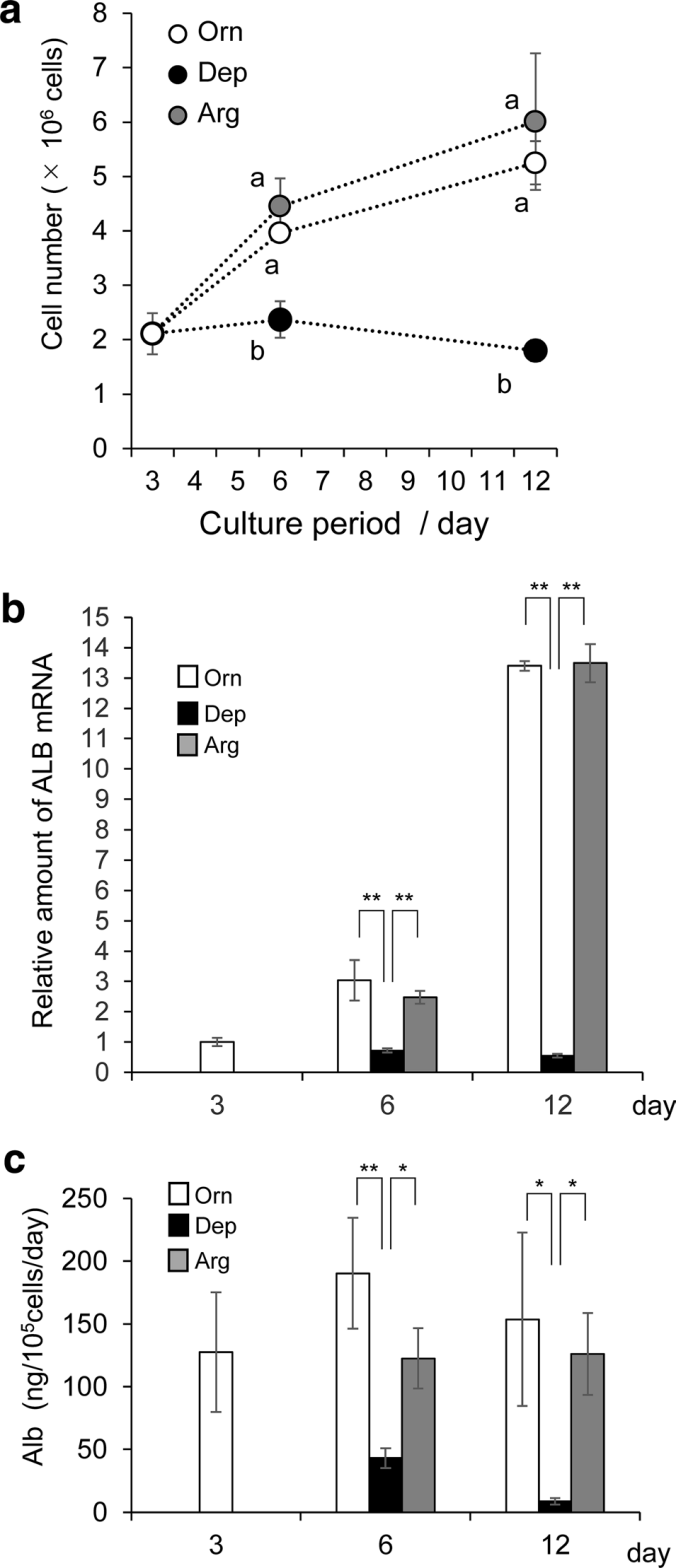
Comparison of cell growth and albumin synthesis under the three culture conditions. **a** Cell growth under the three medium conditions (white: Orn, black: Dep, gray: Arg) (Cells: FLC-4) was analyzed. All values in this figure are shown as the average of three samples (*n* = 3). For the three groups (Orn, Arg, and Dep) of samples from the same day (days 6 or 12), significance was analyzed by one-way ANOVA. The data for cell numbers on day 6 (*F* = 27.81, *P* = 0.000923) and day 12 (*F* = 25.62, *P* = 0.001152) revealed significant differences among the three groups. Alphabetical marks indicate significant difference between groups (“a” vs “b”: with significance set to *P* < 0.01). **b** Quantification for albumin mRNA in FLC-4 cells via qPCR analysis. The value from day 3 in ASF104N medium was used as a reference for the other days and media. For the three groups of samples on the same day (days 6 or 12), significance was analyzed by one-way ANOVA. The data for *ALB* on day 6 (*F* = 26.3, *P* = 0.00107) and day 12 (*F* = 556.36, *P* < 0.0001) revealed significant differences among the three groups. Asterisks indicate the results of comparisons between two groups by Tukey’s HSD test (**P < 0.01). **c** Quantification of albumin protein in medium by ELISA. For the three groups of samples from the same day (days 6 or 12), statistical significance was analyzed by one-way ANOVA. The data for albumin protein levels at day 6 (*F* = 18.89, *P* = 0.002574) and day 12 (*F* = 9.12, *P* = 0.015165) revealed significant differences among the three groups. Asterisks indicate the results of comparisons between two groups by Tukey’s HSD test (*P < 0.05, **P < 0.01)

Subsequently, cell growth and albumin production were evaluated in the three different medium conditions (Orn, Arg, and Dep) (Fig. 3a). In terms of cell growth and albumin production, there were no significant differences between the Orn and Arg conditions after the medium was changed on day 3. However, significant differences were observed under Dep conditions as cell growth was severely arrested on days 3‒12, and decreased slightly. Albumin production in cells cultured under Dep conditions on days 6 and 12 was significantly lower at the mRNA level, compared to that in cells cultured under Orn and Arg conditions, as was the amount of albumin protein quantified by ELISA (Fig. 3b, c). These results confirmed that FLC-4 cells require either arginine or ornithine, which are amino acids related to the urea cycle, and that a lack of both amino acids significantly affects albumin production and cell growth.

### Expression of liver-enriched transcriptional regulatory factors under the three medium conditions

**Fig. 4:**
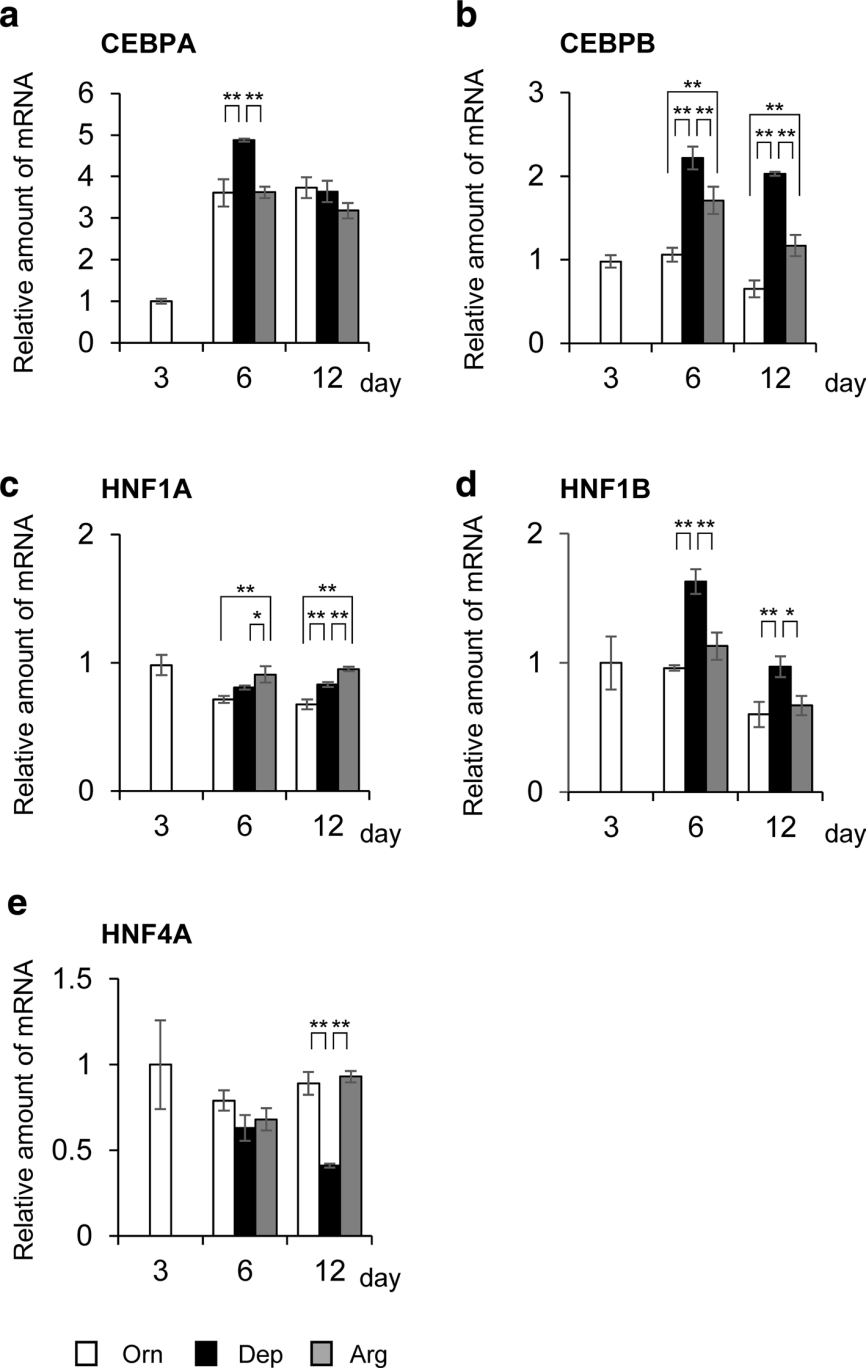
Analysis of the transcription levels of liver-enriched transcription factors. Quantification of mRNA levels under three medium conditions (white: Orn, black: Dep, gray: Arg). The mRNA levels of five liver-enriched transcription factors {*CEBPA* (**a**), *CEBPB* (**b**), *HNF1A* (**c**), *HNF1B* (**d**), and *HNF4A* (**e**)} are indicated. The corresponding gene symbol is indicated at the top left of each graph. The value at day 3 in Orn medium was used as a reference for the other days and medium conditions. All values in this figure are shown as the average of three samples (*n* = 3). Error bars indicate standard deviation. For the three groups from different medium conditions (Orn, Arg, and Dep) in samples from the same day (days 6 or 12), statistically significance was analyzed by one-way ANOVA. The data for *CEBPA* at day 12 (*F* = 4.8, *P* = 0.056896) and *HNF4A* at day 6 (*F* = 4.57, *P* = 0.062241) exhibited no significant differences among the three groups (*P* > 0.05). Other data sets showed significance among the three groups {*CEBPA*: day 6; (*F* = 37.15, *P* = 0.000417)} {*CEBPB*: day 6; (*F* = 57.49, *P* = 0.000122), day 12; (*F* = 163.05, *P* < 0.0001)} {*HNF1A*: day 6; (*F* = 17.62, *P* = 0.003080), day 12; (*F* = 79.88, *P* < 0.0001)} {*HNF1B*: day 6; (*F* = 52.28, *P* = 0.000160), day 12; (*F* = 15.93, *P* = 0.003980)}{*NHF4A*: day 12; (*F* = 126.36, *P* < 0.0001)}. Asterisks represent the results of comparisons between two groups via Tukey’s HSD test (*P < 0.05, **P < 0.01)

Several liver-enriched transcription factors, in particular the members of C/EBP and HNF families, play critical roles in the transcriptional regulation of liver-specific genes, including albumin [13]. To understand the effects of amino acids involved in urea cycle on FLC-4 cells, the mRNA levels of five liver-enriched transcription factors (*CEBPA*, *CEBPB*, *HNF1A, HNF1B,* and *HNF4A*) were evaluated by qPCR (Fig. 4). On comparing gene expression under the three medium conditions, all genes showed significant differences in either or both culture periods by one-way ANOVA (*n* = 3, *F* ≥ 15.93, *P* ≤ 0.003980). Most exhibited significant increases when cultured under Dep conditions, except for *HNF4A*. The mRNA levels of *CEBPA* at day 6 and *HNF1B* (both days 6 and 12) were elevated significantly in the Dep condition (Fig. 4a, d). The mRNA level of *HNF4A* at day 12 was decreased significantly in the Dep condition (Fig. 4e). For *CEBPB* and *HNF1A*, significant differences were also observed between Orn and Arg conditions (Fig. 4b, c). In these genes, transcriptional levels under Arg conditions were higher than those observed under Orn conditions (Fig. 4c). These results suggest that arginine and ornithine do not play identical roles with respect to regulating the expression of the five liver-enriched transcription factors. Among these five transcription factor genes, *CEBPB* at day 12 exhibited remarkable differences under all three medium conditions (Orn vs Arg; 1.8-fold, Orn vs Dep; 3-fold). Therefore, in subsequent analyses, we analyzed C/EBPβ, focusing on day 12.

### Changes in C/EBPβ protein levels under the three medium conditions

**Fig. 5:**
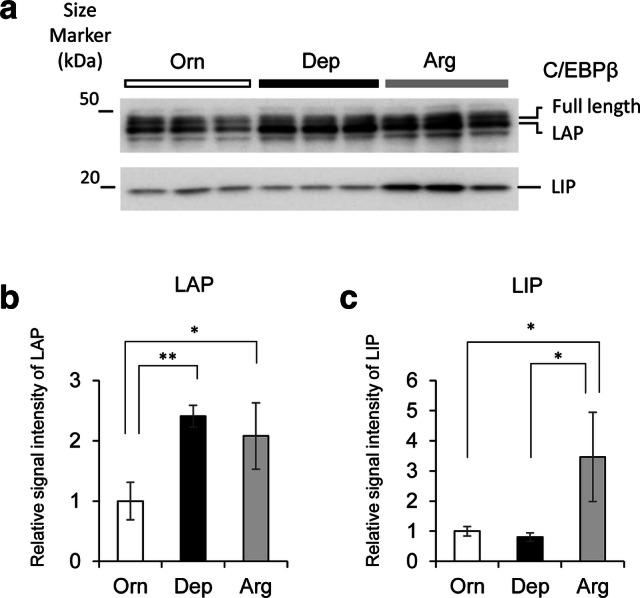
Western blotting for C/EBPβ under the three medium conditions. Western blotting for C/EBPβ was performed on day 12 lysates of cultured cells grown under Orn, Arg, or Dep conditions (**a**). The lane labels indicate the different medium conditions (shown at the top of gel image). Three independent culture samples were tested for each medium condition. Equal amounts of protein (10 μg) were loaded into each well. Semi-quantitative analyses of C/EBPβ isoform expression based on comparison of signal intensity for LAP (**b**) or LIP (**c**) bands are shown. The average band intensities of the three lanes were compared. The values for samples from the Orn medium condition were used as a reference for the other medium conditions. Error bars indicate standard deviation. For the three groups from different medium conditions (Orn, Arg, and Dep), statistical significance was analyzed by one-way ANOVA. Both data sets revealed statistically significant differences among the three groups {LAP: (*F* = 11.21, *P* = 0.009410), LIP: (*F* = 8.9, *P* = 0.016022)}. Asterisks indicate the results of comparisons between two groups by Tukey’s HSD test (*P < 0.05, **P < 0.01)

C/EBPβ is a critical transcription factor involved in the regulation of cell growth and liver-specific protein synthesis, including albumin in hepatocytes and HepG2 cells [13, 23–27]. Three major isoforms (46, 42, and 20 kDa) of C/EBPβ are produced from a single mRNA via the use of different translation start sites. Among these, the 20 kDa form exerts a negative regulatory effect on target gene transcription, and is termed the liver-enriched transcriptional inhibitory protein (LIP) [28]. Hence, we confirmed the generation of the three major isoforms of C/EBPβ protein under the three medium conditions at day 12 by western blotting. The results of western blot analysis showed that immunopositive signals related to these three isoforms were detected under all medium conditions (Fig. 5a). The main signal was observed at a molecular weight of 42 kDa, consistent with that of an isoform referred to as liver-enriched transcriptional activator protein (LAP). Compared with the Orn condition, the immunopositive signals were significantly increased under Dep and Arg conditions (Fig. 5b, c). In particular, significantly stronger LIP signals were observed under Arg conditions (3.5-fold greater than those for Orn) (Fig. 5c). These results suggest that the increase in *CEBPB* mRNA levels, as shown in Fig. 4b, indicates elevation in LAP levels under Dep conditions, and an increase in both LAP and LIP isoforms under Arg conditions.

## Discussion

The data regarding albumin synthesis in the present study showed no significant differences between Orn and Arg conditions (Fig. 3b, c). These results could indicate that albumin synthesis in FLC-4 cells requires the presence of sufficient amounts of ornithine or arginine. However, the results of qPCR for liver-enriched transcription factors were not identical between Orn and Arg medium conditions (Fig. 4). Furthermore, the levels of C/EBPβ and its isoforms differed between Orn and Arg medium conditions (Fig. 5). Multiple liver-enriched transcription factors, including HNFs, C/EBPs, and combinations of them, are related to the expression of liver-specific proteins [13, 25, 29, 30]. Moreover, non-essential amino acids, including arginine, often play various roles in animals [31]. The results of this study suggest that the contributions of the two amino acids studied are not identical in FLC-4 cells, and that they can elicit different cellular responses. Albumin expression is regulated by various signals and exhibits strong homeostasis at the individual level [7–12]. The differences between medium conditions for Orn and Arg in this study might have been within the range of homeostatic maintenance at the cellular level. In our previous studies using RFB, the efficiency of liver-specific protein synthesis varied depending on the culture medium used. These studies used an arginine concentration of 200 mg/L (for IS-RPMI; examined fibrinogen production) [4] and 581.45 mg/mL (for E-RDF; examined albumin production) (Online Resource), respectively in media. Furthermore, it has been reported that three-dimensional culture affects gene expression status in FLC-4 cells [19]. To clarify the differences between this report and previous RFB studies, evaluation of three-dimensional culture conditions and higher levels of arginine represent future areas of study.

Although there appeared to be no difference between arginine and ornithine with respect to albumin production, the deficiency of both amino acids dramatically reduced albumin production. This result suggest that the absence of both ornithine and arginine in the amino acid composition of cell culture medium is important for albumin expression. Another finding was the relationship between ornithine depletion and polyamine synthesis in Dep conditions. Two other research groups have previously concluded that the mechanism underlying the positive role of ornithine in albumin synthesis is related to polyamine synthesis, because treatment with polyamine synthesis pathway blockers (DFMO or MO) that inhibit the generation of putrescine from ornithine abrogates ornithine-induced enhancement of albumin synthesis [7, 16, 17]. However, these authors did not demonstrate any direct evidence that blocking polyamine synthesis mimics ornithine depletion. In our study, using Dep (– ornithine/– arginine) medium and FLC-4 cells, we were able to create actual ornithine-deficient conditions, and analyzed the effects on albumin synthesis. Albumin expression was markedly reduced in FLC-4 cells cultured in Dep medium, although polyamine starvation did not occur (Figs. 1, 3). These results suggest that DFMO (or MO) addition and actual ornithine deficiency represent different conditions. Thus, the results also suggest that ornithine plays a role in the expression of *ALB,* besides acting as a source for polyamine synthesis. Maintaining cellular polyamine levels is critical for a variety of cellular processes [32, 33]. Therefore, in Dep medium, it is thought that the minimal amounts of arginine and ornithine are probably supplied as metabolites of other amino acids derived from the medium, or result from intracellular protein degradation. In experiments in rats, the Michaelis constants of two enzymes employing ornithine as a substrate were shown. The Michaelis constant of ornithine decarboxylase (ODC), which produces putrescine from ornithine, is smaller than that of ornithine transcarbamylase (OTC), one of the key enzymes involved in the urea cycle [14, 34]. This is also the case in FLC-4 cells; lower levels of intracellular ornithine might be occupied by ODC to maintain the polyamine levels.

Insufficient amounts of both ornithine and arginine possibly recreate the effects of clinically severe malnutrition. Dep medium contains a sufficient carbon energy source, but insufficient amino acid components. In other words, it is thought that the Dep condition used in this study can mimic severe nutritional stress related to insufficient protein intake in culture cell lines, thereby mimicking disorders such as Kwashiorkor. Thus, the mechanisms underlying the development of hypoalbuminemia due to insufficient protein intake could be explained not only as representing defective protein synthesis (translation), which originates from deficiencies involving the amino acid components of albumin protein, but also as a result of affected transcription.

## Electronic supplementary material

Below is the link to the electronic supplementary material.

Supplementary file1 (PDF 159 kb)
