# Supplementary material for: Investigation of the effects of urea cycle amino acids on the expression of ALB and CEBPB in the human hepatocellular carcinoma cell line FLC-4
Source: Hum Cell. 2020 May 30;33(3):590–8. doi: 10.1007/s13577-020-00383-1 (PMC7324429; doi:10.1007/s13577-020-00383-1)
Supplement: Supplementary file 1 — Supplementary file1 (PDF 159 kb) [file 13577_2020_383_MOESM1_ESM.pdf]

Appendix

Methods

Radial flow bioreactor culture

A radial flow bioreactor (RFB) system with a 5 mL size radial-flow chamber (Able, Tokyo, Japan) filled with hydroxyapatite ceramic beads (APACERAM-AX, HOYA-PENTAX, Tokyo, Japan) was used in this study. FLC-4 cells (1.5×10^7 cells) maintained in ASF104N medium (Ajinomoto Co., Inc.) were transferred to the RFB at time 0. The culture medium [ASF104N (+ornithine/–arginine)] was replaced with E-RDF (–ornithine/+arginine) (KYOKUTO PHARMACEUTICAL INDUSTRIAL CO., LTD, Tokyo, Japan) on day 14. Cells were cultured for 14 days in each medium for a total culture period of 28 days. In the latter half of each culture period, 50% (50 mL) or the entire (100 mL) volume of the medium in the RFB was renewed every 2–3 days. To determine the albumin concentration in the medium, aliquots of medium were collected 12 times from a reservoir bottle throughout the culture period. The amount of albumin secreted into the medium was determined by ELISA, as described in the main text.

Results

Albumin production in RFB

The time course for albumin secreted into the RFB medium was analyzed (Suppl. Fig. 1). Albumin concentration gradually increased during the initial 14 days in ASF104N medium. On day 14, the medium [ASF104N (+ornithine/–arginine)] was replaced with E-RDF (–ornithine/+arginine). After this, albumin concentration in the culture medium of the RFB was dramatically elevated. Albumin concentrations in E-RDF medium increased approximately two 2-fold within five days after medium replacement.

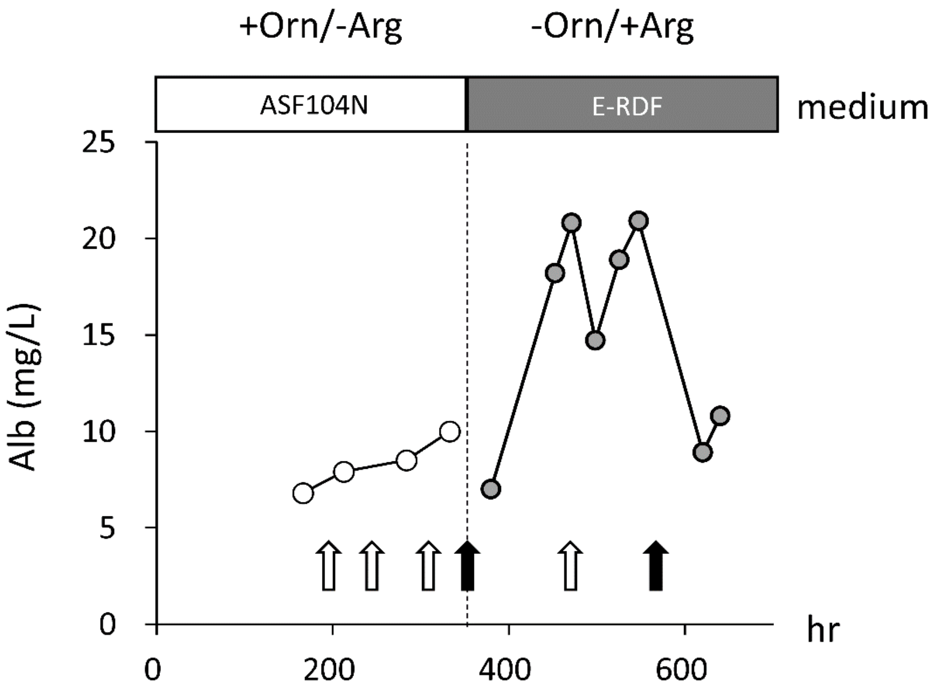

Fig. Time course of albumin secretion from FLC-4 cells cultured in RFB.

FLC-4 cells (1.5 × 10^7 cells) maintained in ASF104N were seeded into an RFB (5-mL). The culture medium, ASF104N (+ornithine/–arginine), was replaced with E-RDF (–ornithine/+arginine) on day 14 (dashed line). Aliquots of the circulating medium were collected at appropriate intervals, and the levels of secreted albumin were determined. Albumin concentrations in ASF104N (white mark) and E-RDF conditions (black mark) were plotted. Arrows in the graph indicate points of medium exchange (black arrow: whole culture volume exchanged, white arrow: half culture volume exchanged).
